# Supplementary material for: Mapping the evolution of fertility support policies in China: A content and instrumental analysis
Source: PLoS One. 2025 Oct 9;20(10):e0332137. doi: 10.1371/journal.pone.0332137 (PMC12510515; doi:10.1371/journal.pone.0332137)
Supplement: S1 Appendix — (ZIP) [file pone.0332137.s001.zip › S1 Appendix. 226 original policy documents/167-中华人民共和国人口与计划生育法(2021修正).doc]

中华人民共和国人口与计划生育法(2021修正)

**发布部门：** [全国人大常委会](https://www.pkulaw.com/law/chl?Aggs.IssueDepartment=201) [C:\email\index](../../../../C:/email/index)

**发文字号：**中华人民共和国主席令第96号

**发布日期：**2021.08.20

**实施日期：**2021.08.20

**时效性：** [现行有效](https://www.pkulaw.com/law/chl?Aggs.TimelinessDic=01)

**效力级别：** [法律](https://www.pkulaw.com/law/chl?Aggs.EffectivenessDic=XA0101)

**法规类别：** [人口计划管理](https://www.pkulaw.com/law/chl?Aggs.Category=09701)

[2015-2021编注](https://www.pkulaw.com/history/compare/编注-4026.html) [2001-2015编注](https://www.pkulaw.com/history/compare/编注-998.html) [C:\Help\more-service-guide.html - feature-8](../../../../C:/Help/more-service-guide.html#feature-8)

中华人民共和国人口与计划生育法

　　（2001年12月29日第九届全国人民代表大会常务委员会第二十五次会议通过　根据2015年12月27日第十二届全国人民代表大会常务委员会第十八次会议《[关于修改〈中华人民共和国人口与计划生育法〉的决定](https://www.pkulaw.com/chl/345b292ebcac3269bdfb.html)》第一次修正　根据2021年8月20日第十三届全国人民代表大会常务委员会第三十次会议《[关于修改〈中华人民共和国人口与计划生育法〉的决定》第二次修正）](https://www.pkulaw.com/chl/345b292ebcac3269bdfb.html)

目录

　　第一章　总则
　　第二章　人口发展规划的制定与实施
　　第三章　生育调节
　　第四章　奖励与社会保障
　　第五章　计划生育服务
　　第六章　法律责任
　　第七章　附则

第一章　总则

　　第一条　为了实现人口与经济、社会、资源、环境的协调发展，推行计划生育，维护公民的合法权益，促进家庭幸福、民族繁荣与社会进步，根据[宪法](https://www.pkulaw.com/chl/7c7e81f43957c58bbdfb.html)，制定本法。

　　第二条　我国是人口众多的国家，实行计划生育是国家的基本国策。

　　国家采取综合措施，调控人口数量，提高人口素质，推动实现适度生育水平，优化人口结构，促进人口长期均衡发展。

　　国家依靠宣传教育、科学技术进步、综合服务、建立健全奖励和社会保障制度，开展人口与计划生育工作。

　　第三条　开展人口与计划生育工作，应当与增加妇女受教育和就业机会、增进妇女健康、提高妇女地位相结合。

　　第四条　各级人民政府及其工作人员在推行计划生育工作中应当严格依法行政，文明执法，不得侵犯公民的合法权益。

　　卫生健康主管部门及其工作人员依法执行公务受法律保护。

　　第五条　国务院领导全国的人口与计划生育工作。

　　地方各级人民政府领导本行政区域内的人口与计划生育工作。

　　第六条　国务院卫生健康主管部门负责全国计划生育工作和与计划生育有关的人口工作。

　　县级以上地方各级人民政府卫生健康主管部门负责本行政区域内的计划生育工作和与计划生育有关的人口工作。

　　县级以上各级人民政府其他有关部门在各自的职责范围内，负责有关的人口与计划生育工作。

　　第七条　工会、共产主义青年团、妇女联合会及计划生育协会等社会团体、企业事业组织和公民应当协助人民政府开展人口与计划生育工作。

　　第八条　国家对在人口与计划生育工作中作出显著成绩的组织和个人，给予奖励。

第二章　人口发展规划的制定与实施

　　第九条　国务院编制人口发展规划，并将其纳入国民经济和社会发展计划。

　　县级以上地方各级人民政府根据全国人口发展规划以及上一级人民政府人口发展规划，结合当地实际情况编制本行政区域的人口发展规划，并将其纳入国民经济和社会发展计划。

　　第十条　县级以上各级人民政府根据人口发展规划，制定人口与计划生育实施方案并组织实施。

　　县级以上各级人民政府卫生健康主管部门负责实施人口与计划生育实施方案的日常工作。

　　乡、民族乡、镇的人民政府和城市街道办事处负责本管辖区域内的人口与计划生育工作，贯彻落实人口与计划生育实施方案。

　　第十一条　人口与计划生育实施方案应当规定调控人口数量，提高人口素质，推动实现适度生育水平，优化人口结构，加强母婴保健和婴幼儿照护服务，促进家庭发展的措施。

　　第十二条　村民委员会、居民委员会应当依法做好计划生育工作。

　　机关、部队、社会团体、企业事业组织应当做好本单位的计划生育工作。

　　第十三条　卫生健康、教育、科技、文化、民政、新闻出版、广播电视等部门应当组织开展人口与计划生育宣传教育。

　　大众传媒负有开展人口与计划生育的社会公益性宣传的义务。

　　学校应当在学生中，以符合受教育者特征的适当方式，有计划地开展生理卫生教育、青春期教育或者性健康教育。

　　第十四条　流动人口的计划生育工作由其户籍所在地和现居住地的人民政府共同负责管理，以现居住地为主。

　　第十五条　国家根据国民经济和社会发展状况逐步提高人口与计划生育经费投入的总体水平。各级人民政府应当保障人口与计划生育工作必要的经费。

　　各级人民政府应当对欠发达地区、少数民族地区开展人口与计划生育工作给予重点扶持。

　　国家鼓励社会团体、企业事业组织和个人为人口与计划生育工作提供捐助。

　　任何单位和个人不得截留、克扣、挪用人口与计划生育工作费用。

　　第十六条　国家鼓励开展人口与计划生育领域的科学研究和对外交流与合作。

第三章　生育调节

　　第十七条　公民有生育的权利，也有依法实行计划生育的义务，夫妻双方在实行计划生育中负有共同的责任。

　　第十八条　国家提倡适龄婚育、优生优育。一对夫妻可以生育三个子女。

　　符合法律、法规规定条件的，可以要求安排再生育子女。具体办法由省、自治区、直辖市人民代表大会或者其常务委员会规定。

　　少数民族也要实行计划生育，具体办法由省、自治区、直辖市人民代表大会或者其常务委员会规定。

　　夫妻双方户籍所在地的省、自治区、直辖市之间关于再生育子女的规定不一致的，按照有利于当事人的原则适用。

　　第十九条　国家创造条件，保障公民知情选择安全、有效、适宜的避孕节育措施。实施避孕节育手术，应当保证受术者的安全。

　　第二十条　育龄夫妻自主选择计划生育避孕节育措施，预防和减少非意愿妊娠。

　　第二十一条　实行计划生育的育龄夫妻免费享受国家规定的基本项目的计划生育技术服务。

　　前款规定所需经费，按照国家有关规定列入财政预算或者由社会保险予以保障。

　　第二十二条　禁止歧视、虐待生育女婴的妇女和不育的妇女。

　　禁止歧视、虐待、遗弃女婴。

第四章　奖励与社会保障

　　第二十三条　国家对实行计划生育的夫妻，按照规定给予奖励。

　　第二十四条　国家建立、健全基本养老保险、基本医疗保险、生育保险和社会福利等社会保障制度，促进计划生育。

　　国家鼓励保险公司举办有利于计划生育的保险项目。

　　第二十五条　符合法律、法规规定生育子女的夫妻，可以获得延长生育假的奖励或者其他福利待遇。

　　国家支持有条件的地方设立父母育儿假。

　　第二十六条　妇女怀孕、生育和哺乳期间，按照国家有关规定享受特殊劳动保护并可以获得帮助和补偿。国家保障妇女就业合法权益，为因生育影响就业的妇女提供就业服务。

　　公民实行计划生育手术，享受国家规定的休假。

　　第二十七条　国家采取财政、税收、保险、教育、住房、就业等支持措施，减轻家庭生育、养育、教育负担。

　　第二十八条　县级以上各级人民政府综合采取规划、土地、住房、财政、金融、人才等措施，推动建立普惠托育服务体系，提高婴幼儿家庭获得服务的可及性和公平性。

　　国家鼓励和引导社会力量兴办托育机构，支持幼儿园和机关、企业事业单位、社区提供托育服务。

　　托育机构的设置和服务应当符合托育服务相关标准和规范。托育机构应当向县级人民政府卫生健康主管部门备案。

　　第二十九条　县级以上地方各级人民政府应当在城乡社区建设改造中，建设与常住人口规模相适应的婴幼儿活动场所及配套服务设施。

　　公共场所和女职工比较多的用人单位应当配置母婴设施，为婴幼儿照护、哺乳提供便利条件。

　　第三十条　县级以上各级人民政府应当加强对家庭婴幼儿照护的支持和指导，增强家庭的科学育儿能力。

　　医疗卫生机构应当按照规定为婴幼儿家庭开展预防接种、疾病防控等服务，提供膳食营养、生长发育等健康指导。

　　第三十一条　在国家提倡一对夫妻生育一个子女期间，自愿终身只生育一个子女的夫妻，国家发给《独生子女父母光荣证》。

　　获得《独生子女父母光荣证》的夫妻，按照国家和省、自治区、直辖市有关规定享受独生子女父母奖励。

　　法律、法规或者规章规定给予获得《独生子女父母光荣证》的夫妻奖励的措施中由其所在单位落实的，有关单位应当执行。

　　在国家提倡一对夫妻生育一个子女期间，按照规定应当享受计划生育家庭老年人奖励扶助的，继续享受相关奖励扶助，并在老年人福利、养老服务等方面给予必要的优先和照顾。

　　第三十二条　获得《独生子女父母光荣证》的夫妻，独生子女发生意外伤残、死亡的，按照规定获得扶助。县级以上各级人民政府建立、健全对上述人群的生活、养老、医疗、精神慰藉等全方位帮扶保障制度。

　　第三十三条　地方各级人民政府对农村实行计划生育的家庭发展经济，给予资金、技术、培训等方面的支持、优惠；对实行计划生育的贫困家庭，在扶贫贷款、以工代赈、扶贫项目和社会救济等方面给予优先照顾。

　　第三十四条　本章规定的奖励和社会保障措施，省、自治区、直辖市和设区的市、自治州的人民代表大会及其常务委员会或者人民政府可以依据本法和有关法律、行政法规的规定，结合当地实际情况，制定具体实施办法。

第五章　计划生育服务

　　第三十五条　国家建立婚前保健、孕产期保健制度，防止或者减少出生缺陷，提高出生婴儿健康水平。

　　第三十六条　各级人民政府应当采取措施，保障公民享有计划生育服务，提高公民的生殖健康水平。

　　第三十七条　医疗卫生机构应当针对育龄人群开展优生优育知识宣传教育，对育龄妇女开展围孕期、孕产期保健服务，承担计划生育、优生优育、生殖保健的咨询、指导和技术服务，规范开展不孕不育症诊疗。

　　第三十八条　计划生育技术服务人员应当指导实行计划生育的公民选择安全、有效、适宜的避孕措施。

　　国家鼓励计划生育新技术、新药具的研究、应用和推广。

　　第三十九条　严禁利用超声技术和其他技术手段进行非医学需要的胎儿性别鉴定；严禁非医学需要的选择性别的人工终止妊娠。

第六章　法律责任

　　第四十条　违反本法规定，有下列行为之一的，由卫生健康主管部门责令改正，给予警告，没收违法所得；违法所得一万元以上的，处违法所得二倍以上六倍以下的罚款；没有违法所得或者违法所得不足一万元的，处一万元以上三万元以下的罚款；情节严重的，由原发证机关吊销执业证书；构成犯罪的，依法追究刑事责任：

　　（一）非法为他人施行计划生育手术的；

　　（二）利用超声技术和其他技术手段为他人进行非医学需要的胎儿性别鉴定或者选择性别的人工终止妊娠的。

　　第四十一条　托育机构违反托育服务相关标准和规范的，由卫生健康主管部门责令改正，给予警告；拒不改正的，处五千元以上五万元以下的罚款；情节严重的，责令停止托育服务，并处五万元以上十万元以下的罚款。

　　托育机构有虐待婴幼儿行为的，其直接负责的主管人员和其他直接责任人员终身不得从事婴幼儿照护服务；构成犯罪的，依法追究刑事责任。

　　第四十二条　计划生育技术服务人员违章操作或者延误抢救、诊治，造成严重后果的，依照有关法律、行政法规的规定承担相应的法律责任。

　　第四十三条　国家机关工作人员在计划生育工作中，有下列行为之一，构成犯罪的，依法追究刑事责任；尚不构成犯罪的，依法给予处分；有违法所得的，没收违法所得：

　　（一）侵犯公民人身权、财产权和其他合法权益的；

　　（二）滥用职权、玩忽职守、徇私舞弊的；

　　（三）索取、收受贿赂的；

　　（四）截留、克扣、挪用、贪污计划生育经费的；

　　（五）虚报、瞒报、伪造、篡改或者拒报人口与计划生育统计数据的。

　　第四十四条　违反本法规定，不履行协助计划生育管理义务的，由有关地方人民政府责令改正，并给予通报批评；对直接负责的主管人员和其他直接责任人员依法给予处分。

　　第四十五条　拒绝、阻碍卫生健康主管部门及其工作人员依法执行公务的，由卫生健康主管部门给予批评教育并予以制止；构成违反治安管理行为的，依法给予治安管理处罚；构成犯罪的，依法追究刑事责任。

　　第四十六条　公民、法人或者其他组织认为行政机关在实施计划生育管理过程中侵犯其合法权益，可以依法申请行政复议或者提起行政诉讼。

第七章　附则

　　第四十七条　中国人民解放军和中国人民武装警察部队执行本法的具体办法，由中央军事委员会依据本法制定。

　　第四十八条　本法自2002年9月1日起施行。
